# European prognosis evaluation of early-stage lung adenocarcinoma patterns after lobectomy versus segmentectomy based on clinical stage settings

**DOI:** 10.1016/j.xjon.2026.101687

**Published:** 2026-02-18

**Authors:** Lukadi Joseph Lula, Lin Huang, Clara Forcada Barreda, Rita Costa, Matic Domjan, Aimée J.P.M. Franssen, Crt Jasovic, Mohamed Rebei, Beatrice Trabalza Marinucci, Rebecca Weedle, Guillermo Rodriguez, Emrah Gökay Özgür, Kiarash Ghasemi, Jack Whooley, Erino Angelo Rendina, Ronan Ryan, Vincent Young, Gülnaz Nural Bekiroglu, Karen Redmond, Erik R. de Loos, Gerard J. Fitzmaurice, Ciprian Bolca, Adelino Leite Moreira, Cornel Savu, Antonio D'Andrilli, Tomaz Stupnik, Alessio Vincenzo Mariolo, Jozsef Furak, Marcelo Jimenez, Alessandro Brunelli, René Horsleben Petersen

**Affiliations:** a Service of Thoracic Surgery, Department of General Surgery, Sherbrooke University, Charles LeMoyne Hospital, Longueuil, Quebec, Canada; b The Thoracic Surgery Department, Institute of Pneumology Marius Nasta, Bucharest, Romania; c Department of Thoracic Surgery, Shanghai Chest Hospital, Shanghai Jiao Tong University School of Medicine, Shanghai, China; d Department of Cardiothoracic Surgery, Copenhagen University Hospital, Rigshospitalet, Copenhagen, Denmark; e Service of Thoracic Surgery, Department of General Surgery, Salamanca University Hospital, Salamanca Institute of Biomedical Research (IBSAL), Salamanca, Spain; f Department of Cardiothoracic Surgery, Centro Hospitalar Universitário São João, Porto, Portugal; g Department of Thoracic Surgery, University Medical Center, Ljubljana, Slovenia; h Division of General Thoracic Surgery, Department of Surgery, Zuyderland Medical Center, Ljubljana, Slovenia; i Thoracic Department, Curie-Montsouris Institute, Institut Mutualiste Montsouris, Paris, France; j Department of Thoracic Surgery, Sant Andrea Hospital, Sapienza University of Rome, Rome, Italy; k Department of Cardiothoracic Surgery, St James's Hospital, Dublin, Republic of Ireland; l Department of Cardiothoracic Surgery, Mater Misericordiae University Hospital, Dublin, Republic of Ireland; m Faculty of Medicine, Department of Biostatistics, Marmara University, Istanbul, Turkey; n Department of Thoracic Surgery, University of Szeged, Szeged, Hungary; o School of Medicine, Trinity St James's Cancer Institute, St James's Hospital, Dublin, Republic of Ireland; p Department of Thoracic Surgery, St James's University Hospital, Leeds, United Kingdom

**Keywords:** lobectomy, lung adenocarcinoma, segmentectomy, patterns, prognosis

## Abstract

**Objectives:**

To investigate the prognosis of peripheral early-stage lung adenocarcinoma patterns treated by lobectomy or segmentectomy.

**Methods:**

Retrospective multicentric cohort of patients with cT1a-bN0M0 lung adenocarcinoma who underwent lobectomy or segmentectomy with systematic lymph node dissection in 10 European centers (one per country) from 2015 to 2021. Overall survival (OS), disease-free survival (DFS), and lung cancer–specific death (LCSD) between both groups were assessed in entire dataset and in dataset of histologic aggressive patterns, before and after propensity score-matching (PSM). Prognostic risk factors were analyzed using parsimonious model Cox regression. Recurrences were assessed by linearized risks.

**Results:**

Lobectomy and segmentectomy were performed in 1029 (73.1%) and 377 (26.8%) patients, respectively. In total, 427 (30.3%) patients had at least 1 histologic aggressive (micropapillary or solid) pattern, and 88 patients (20.7%) underwent segmentectomy. OS, DFS, and LCSD rates were similar between patients who underwent lobectomy or segmentectomy, in both datasets, before and after PSM. In aggressive dataset, PSM, 5-year OS rates were lobectomy 88.0% (95% CI, 80.9-95.7%), segmentectomy 89.1% (95% CI, 82.2-96.6%), *P* = .8; 5-year DFS rates were lobectomy 79.8% (95% CI, 70.8-89.8%), segmentectomy 80.6% (95% CI, 71.6-90.6%), *P* = .6; and 5-year LCSD rates were lobectomy 6.0%, segmentectomy 7.8%, *P* = .8. Locoregional recurrence was not superior in patients who underwent segmentectomy in entire dataset (linearized risks: lobectomy 0.078, segmentectomy 0.073) and in aggressive dataset (linearized risks: lobectomy 0.036, segmentectomy 0.011) only in the unmatched cohorts. Aggressive histologic patterns impacted on only LCSD, and only when they were dominant.

**Conclusions:**

Segmentectomy seems comparable to lobectomy for patients with peripheral cT1a-bN0M0 lung adenocarcinoma even in case of histologic aggressive patterns.

**Figure:**
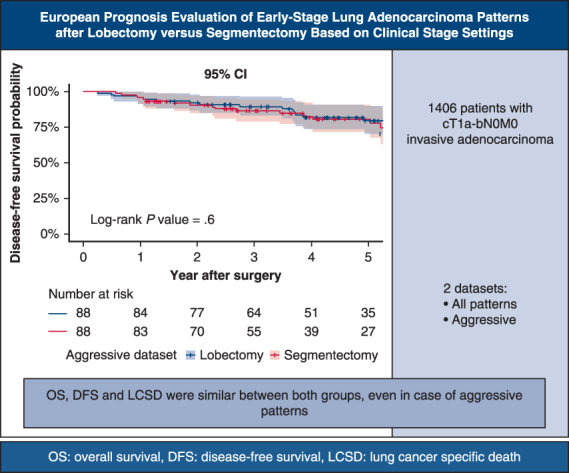
DFS of the matched aggressive dataset. *OS*, Overall survival; *DFS*, disease-free survival; *LCSD*, lung cancer–specific death.

**Central Message**

We assessed impact of histologic patterns on oncologic outcomes according to lung resection extent. Oncologic outcomes were similar between lobectomy versus segmentectomy, even in aggressive patterns.

**Perspective**

Segmentectomy with systematic lymph node dissection can be considered for patients with suspected or confirmed aggressive histologic adenocarcinoma patterns.

Lung adenocarcinoma, which accounts for roughly one half of all lung cancers, is a highly heterogeneous disease in many respects. The current histologic classification was reviewed in 2021.^1^ Solid and micropapillary patterns are aggressive and can be classified, like others, as dominant or nondominant. They are mainly nondominant, but still impactful^2^, ^3^, ^4^, ^5^ even when their presence is less than 5%,^6^ and not clinically reported. At least one third of patients with clinical early-stage adenocarcinoma present with these aggressive histologic patterns.^3^^,^^4^^,^^6^

These patterns are not only associated with other poor pathologic features and their subsequent worse oncologic outcome, but they are also related to demographic^4^^,^^5^^,^^7^ and clinical characteristics,^4^^,^^8^^,^^9^ which makes them predictable. Most importantly, they seem to be influenced by the quality of resection.^7^ Therefore, an oncologic lung resection with safe margins and systematic lymph node dissection may mitigate their impact. In addition, lung resection provides the entire specimen (tumor and lymph nodes) for a comprehensive diagnosis with greater granularity. This incentivizes a standardized assessment and an individualization of lung cancer treatment. These diagnosis and therapeutic benefits of lung resection can also be achieved by segmentectomy.

In our previous series, we reported that patients with pathologic T1a-bN0M0 adenocarcinoma and histologic aggressive patterns who had undergone segmentectomy did not require a completion lobectomy.^8^ Micropapillary and solid patterns are strongly associated with nodal upstaging, so we aim to investigate the impact of these patterns on oncologic outcome in clinical settings.

## Methods

### Ethics Statement

This study was approved by the Ethics Committee for Clinical Research of Institute of Pneumology Marius Nasta (approval number: 13944/16.06.2023). The approval was either automatically accepted by attending centers or required an additional local approval. Since the study was retrospective and used deidentified data for analyses, patients’ consent was waived.

### Study Cohort

A retrospective European cohort study across 10 centers was performed. Eight of the centers are contributors to the European Society of Thoracic Surgeons registry (Table 1). Data were collected from patients who underwent either lobectomy or segmentectomy with lymph node dissection (according to the European Society of Thoracic Surgeons recommendations)^9^ for peripheral cT1a-bN0M0 (TNM ninth edition) invasive adenocarcinoma between January 2015 and December 2021. Exclusion criteria involved patients with a history of other cancer than non–small cell lung cancer, adenosquamous carcinoma, multiple nodules, no histologic classification, no nodal description, and those who died in the first 3 months.

**Table 1:** Attending centers

Center	Country
Institute of Pneumology Marius Nasta	Romania
Copenhagen University Hospital	Denmark
Salamanca University Hospital	Spain
São João University Hospital	Portugal
University Medical Centre of Ljubljana	Slovenia
Zuyderland Medical Center	The Netherlands
Institut Mutualiste Montsouris	France
Sant’Andrea Hospital	Italy
St James's Hospital	Ireland
University of Szeged	Hungary

The following adenocarcinoma patterns were considered: lepidic, acinar, papillary, solid, and micropapillary. Solid and micropapillary patterns were labeled as aggressive. Adenocarcinoma classification was performed according to the World Health Organization 2021 classification^1^ by dedicated pathologists microscopically in 9 centers and digitally in 1 center. Routine discussion of the classification (by at least 2 pathologists) was only conducted in 3 centers. Maximum standardized uptake value (SUVmax) was categorized according to the classification from Sun and colleagues.^10^ Segmentectomy was performed at the discretion of each center. Peripheral tumors consisted in those located in one-third outer lung.

### Follow-up

Patient management was discussed in multidisciplinary team meetings, held across all centers. Patients were assessed by surgeons shortly after operation, and they were subsequently followed up by oncologists or pulmonologists. Patients were routinely seen every quarter or semester for the first 2 years, after which they were followed up annually. The follow-up period ended in January 2024. Recurrences were categorized as locoregional (in ipsilateral lung: resection margins, hilar or mediastinal lymph nodes, and pleural invasion) or distant (contralateral lung or extrathoracic). Second primary cancers or recurrence were either confirmed (pathologically) or suspected by multidisciplinary team.

### Statistical Methods

Statistical analyses were conducted using R software, version 4.2.2. Discrete random variables were presented as numbers and percentages, whereas continuous random variables were described using median and interquartile range, mean and standard deviation.

Disease-free survival (DFS) was defined as duration from date of intervention to date of recurrence, death, or last follow-up visit. Overall survival (OS) was defined as time from date of intervention to death from any cause or last follow-up visit. Lung cancer–specific death (LCSD) was defined as interval from date of operation to death from lung cancer. We used the cumulative incidence method, considering mortality from any other cause than lung cancer, as a competing risk. The Gray test was used to evaluate the difference between groups.

The χ^2^ and Fisher exact tests were used to compare discrete random variables, whereas the Levene and Student *t* tests were used to assess the equality of variances. The Mann–Whitney *U* test was performed to compare continuous random variables. The Kaplan–Meier method was used to analyze survivals and log-rank test for the comparison.

Follow-up was assessed by number of outcomes/100 patients-year. Parsimonious Cox proportional hazards regression model was performed in both datasets, including clinically relevant variables to assess survival. The model was adjusted to include the interaction of variables that violated proportional hazards assumption with time and those that were significant in univariate analysis. The final model was obtained by using stepwise method. Hazard ratios (HRs) and 95% CIs are presented.

To address a possible residual impact of confounding covariables after regression-based adjustment, we performed propensity score-matching 1:1, with the following variables considered in entire dataset: age, sex, comorbidities, approach, clinical tumor size, pathologic tumor size, and nondominant pattern; and in aggressive pattern dataset: age, sex, and comorbidities were considered. The nearest neighbor method was used with a minimum caliper value 0.2. Standardized mean differences were used to assess the imbalance of covariates in matched groups, and stratified tests were used to compare survivals within these matched groups.

To evaluate proportional hazards assumption, the Schoenfeld residuals test was used when survival curves crossed. We handled multicollinearity by using the parsimonious model and the proportional hazards assumption.

Primary end points of this study were analyses of DFS, OS, and LCSD in entire and aggressive datasets between lobectomy versus segmentectomy. Secondary end points were as follows:

• assessing whether segmentectomy was an independent risk factor for DFS, OS, and LCSD in both datasets;
• comparing locoregional recurrence between lobectomy versus segmentectomy in the 2 datasets;
• assessing whether aggressive dominant or nondominant patterns were independent risk for DFS, OS, and LCSD in entire dataset; and
• assessing other variables that may influence DFS, OS, and LCSD in both datasets.

## Results

In total, 1406 patients with cT1a-bN0M0 invasive lung adenocarcinoma were included. Lobectomy and segmentectomy were respectively performed in 1029 (73.1%) and 377 (26.8%) (Table 2). 427 (30.4%) patients had at least 1 aggressive pattern, and 88 (20.6%) of them underwent segmentectomy (Table 3). The number of outcomes per 100 patients-year was as follows in entire dataset: 5.69 for OS, 3.78 for DFS, and 2.41 for LCSD (Table E1). Segmentectomy did not influence OS, DFS, or LCSD.

**Table 2:** Patient characteristics of the entire dataset

Operation	Before propensity score matching			After propensity score matching			
	Lobectomy, n = 1029	Segmentectomy, n = 377	*P* value	Lobectomy, n = 377	Segmentectomy, n = 377	*P* value	SMD
Sex			.009			.560	0.04
Female	586 (56.9%)	185 (49.1%)		177 (46.9%)	185 (49.1%)		
Male	443 (43.1%)	192 (50.9%)		200 (53.1%)	192 (50.9%)		
Age, y	68 (61-73)	70 (62-74)	<.001	69 (63-74)	70 (62-74)	.647	<0.001
PS			.055			.868	0.03
0	823 (80.1%)	322 (85.4%)		317 (84.1%)	322 (85.4%)		
1	178 (17.3%)	50 (13.3%)		54 (14.3%)	50 (13.3%)		
2	27 (2.6%)	5 (1.3%)		6 (1.6%)	5 (1.3%)		
Comorbidities			.002			.114	0.07
No	217 (21.1%)	71 (18.9%)		68 (18.0%)	72 (19.1%)		
Cardiac	150 (14.6%)	75 (19.9%)		60 (15.9%)	75 (19.9%)		
Respiratory	81 (7.9%)	25 (6.6%)		31 (8.2%)	25 (6.6%)		
Previous lung cancer	11 (1.1%)	10 (2.7%)		3 (0.8%)	10 (2.7%)		
Other	231 (22.5%)	58 (15.4%)		77 (20.5%)	58 (15.4%)		
More than 1	337 (32.8%)	137 (36.4%)		138 (36.6%)	137 (36.3%)		
Smoking status			.207			.136	0.11
Never	242 (23.6%)	72 (19.1%)		95 (25.2%)	72 (19.1%)		
Ex	517 (50.2%)	203 (54.0%)		188 (49.9%)	203 (54.0%)		
Current	270 (26.2%)	101 (26.9%)		94 (24.9%)	101 (49.9%)		
Pack year			.006			.021	0.01
<20	406 (39.5%)	130 (34.5%)		147 (39.0%)	130 (34.5%)		
≥20	534 (51.9%)	228 (60.5%)		196 (52.0)	228 (60.5%)		
Unknown	89 (8.6%)	19 (17.6%)		34 (9.0%)	19 (5.0%)		
Approach			<.001			.148	0.10
Open	310 (30.1%)	160 (42.4%)		152 (40.3%)	160 (42.4%)		
VATS	468 (45.5%)	171 (45.4%)		160 (42.4%)	171 (45.4%)		
RATS	251 (24.4%)	46 (12.2%)		65 (17.3%)	46 (12.2%)		
Molecular mutations			.419			.342	0.02
Not performed	860 (83.6%)	319 (84.6%)		313 (83.0%)	319 (84.6%)		
EGFR	86 (8.4%)	23 (6.1%)		35 (9.3%)	23 (6.1%)		
KRAS	37 (3.6%)	20 (5.3%)		15 (4.0%)	20 (5.3%)		
ALK	10 (1.0%)	2 (0.5%)		5 (1.3%)	2 (0.5%)		
Others	23 (2.2%)	10 (2.7%)		8 (2.1%)	10 (2.7%)		
More than 1	13 (1.2%)	3 (0.8)		1 (0.3%)	3 (0.8%)		
Clinical tumor size			<.001			.630	0.04
≤1 cm	206 (20.0%)	112 (29.7%)		106 (28.1%)	112 (29.7%)		
1< × ≤2 cm	823 (80.0%)	265 (70.3%)		271 (71.9%)	265 (70.3%)		
Pathologic tumor size			.008			1.000	<0.01
≤2 cm	965 (93.8%)	367 (97.3%)		367 (97.3%)	367 (97.3%)		
>2 cm	64 (6.2%)	10 (2.7%)		10 (2.7%)	10 (2.7%)		
Pathologic N			.408			.086	0.04
0	943 (91.6%)	352 (93.4%)		342 (90.7%)	352 (93.4%)		
1	53 (5.2%)	13 (3.4%)		26 (6.9%)	13 (3.4%)		
2	33 (3.2%)	12 (3.2%)		9 (2.4%)	12 (3.2%)		
Dominant			.471			.262	0.12
Aggressive	155 (15.1%)	51 (13.5%)		62 (16.4%)	51 (13.5%)		
Others	874 (84.9%)	326 (86.5%)		315 (83.6%)	326 (86.5%)		
Nondominant			<.001			.285	0.01
Aggressive	204 (19.8%)	43 (11.4%)		35 (9.3%)	43 (11.4%)		
Others	298 (29.0%)	63 (16.7%)		78 (20.7%)	63 (16.7%)		
Non class	527 (51.2%)	271 (71.9%)		264 (70.0%)	271 (71.9%)		
SUVmax			.140			.061	0.19
Not done	148 (14.4%)	65 (17.2%)		54 (14.3%)	65 (17.2%)		
<2.01	216 (21.0%)	93 (24.7%)		75 (19.9%)	93 (24.7%)		
2.01 ≤ × <7.41	393 (38.2%)	139 (36.9%)		141 (37.4%)	139 (36.9%)		
≥7.41	190 (18.4%)	52 (13.8%)		58 (15.4%)	52 (13.8%)		
Unknown	82 (8.0%)	28 (7.4%)		49 (13.05)	28 (7.4%)		
Adjuvant chemotherapy	157 (15.3%)	65 (17.2%)	.370	68 (18.1%)	65 (17.2%)	.761	0.03

*SMD*, Standardized mean difference; *PS*, performance status; *VATS*, video-assisted thoracic surgery; *RATS*, robotic-assisted thoracic surgery; *SUVmax*, maximum standardized uptake value.

**Table 3:** Patient characteristics from the aggressive dataset

Operation	Before propensity score matching			After propensity score matching			
	Lobectomy, n = 339	Segmentectomy, n = 88	*P* value	Lobectomy, n = 88	Segmentectomy, n = 88	*P* value	SMD
Sex			.814			.647	0.07
Female	184 (54.3%)	49 (55.7%)		52 (59.1%)	49 (55.7%)		
Male	155 (45.7%)	39 (44.3%)		36 (40.9%)	39 (44.3%)		
Age, y	66 (60-72)	71 (65-76)	<.001	70.50 (64.25-75.75)	71 (65-76)	.799	0.02
PS			.210			.242	0.07
0	244 (72.0%)	71 (80.7%)		62 (70.5%)	71 (80.7%)		
1	84 (24.8%)	14 (15.9%)		23 (26.1%)	14 (15.9%)		
2	11 (3.2%)	3 (3.4%)		3 (3.4%)	3 (3.4%)		
Comorbidities			.024			.300	0.01
No	45 (13.4%)	9 (10.2%)		10 (11.4%)	9 (10.2%)		
Cardiac	37 (11.0%)	14 (15.9%)		12 (13.6%)	14 (15.9%)		
Respiratory	27 (8.0%)	3 (3.4%)		3 (3.4%)	3 (3.4%)		
Previous lung cancer	4 (1.2%)	5 (5.7%)		0 (0.0%)	5 (5.7%)		
Other	87 (25.8%)	16 (18.2%)		14 (15.9%)	16 (18.2%)		
More than 1	137 (40.6)	41 (46.6%)		49 (55.7%)	41 (46.6%)		
Smoking status			.805			.755	0.07
Never	56 (16.5%)	12 (13.8%)		12 (13.6%)	12 (13.8%)		
Ex	185 (54.6%)	48 (55.2%)		53 (60.2%)	48 (55.2%)		
Current	98 (28.9%)	27 (31.0%)		23 (26.1%)	27 (31.0%)		
Pack year			.772			.730	0.01
<20	109 (32.2%)	25 (28.4%)		28 (31.8%)	25 (28.4%)		
≥20	198 (58.4%)	55 (62.5%)		50 (56.6%)	55 (62.5%)		
Unknown	32 (9.4%)	8 (9.1%)		10 (11.4%)	8 (9.1%)		
Approach			.169			.292	0.16
Open	76 (22.4%)	27 (30.7%)		18 (20.5%)	27 (30.7%)		
VATS	195 (57.5%)	49 (55.7%)		58 (65.9%)	49 (55.7%)		
RATS	68 (20.1%)	12 (13.6%)		12 (13.6%)	12 (13.6%)		
Molecular mutations			.200			.152	0.10
Not performed	270 (79.7%)	69 (78.5%)		74 (84.1%)	69 (78.5%)		
EGFR	32 (9.4%)	5 (5.7%)		6 (6.8%)	5 (5.7%)		
KRAS	16 (4.7%)	10 (11.4%)		3 (3.4%)	10 (11.4%)		
ALK	6 (1.8%)	1 (1.1%)		0 (0.0%)	1 (1.1%)		
Others	13 (3.8%)	2 (2.2%)		5 (5.7%)	2 (2.2%)		
More than 1	2 (0.6%)	1 (1.1.%)		0 (0.0%)	1 (1.1.%)		
Clinical tumor size			.203			.303	0.19
≤1 cm	78 (23.0%)	26 (29.5%)		20 (22.7%)	26 (29.5%)		
1 < × ≤2 cm	261 (77.0%)	62 (70.5%)		68 (77.3%)	62 (70.5%)		
Pathologic tumor size			.80			1.000	<0.001
≤2 cm	314 (92.6%)	86 (97.7%)		86 (97.7%)	86 (97.7%)		
>2 cm	25 (7.4%)	2 (2.3%)		2 (2.3%)	2 (2.3%)		
Pathologic N			.645			1.000	0.03
0	316 (93.2%)	85 (96.7%)		84 (95.4%)	85 (96.7%)		
1	12 (3.5%)	1 (1.1%)		2 (2.3%)	1 (1.1%)		
2	11 (3.2%)	2 (2.2%)		2 (2.3%)	2 (2.2%)		
SUVmax			.395			.605	0.10
Not done	45 (13.3%)	16 (18.2%)		17 (19.3%)	16 (18.2%)		
<2.01	56 (16.5%)	14 (15.9%)		12 (13.6%)	14 (15.9%)		
2.01 ≤ × <7.41	136 (40.1%)	31 (35.2%)		40 (45.5%)	31 (35.2%)		
≥7.41	76 (22.4%)	16 (18.2%)		11 (12.5%)	16 (18.2%)		
Unknown	26 (7.7%)	11 (12.5%)		8 (9.1%)	11 (12.5%)		
Adjuvant chemotherapy	60 (17.7%)	18 (20.5%)	.551	14 (15.9%)	18 (20.5%)	.434	0.16

*SMD*, Standardized mean difference; *PS*, performance status; *VATS*, video-assisted thoracic surgery; *RATS*, robotic-assisted thoracic surgery; *SUVmax*, maximum standardized uptake value.

**Figure 1:**
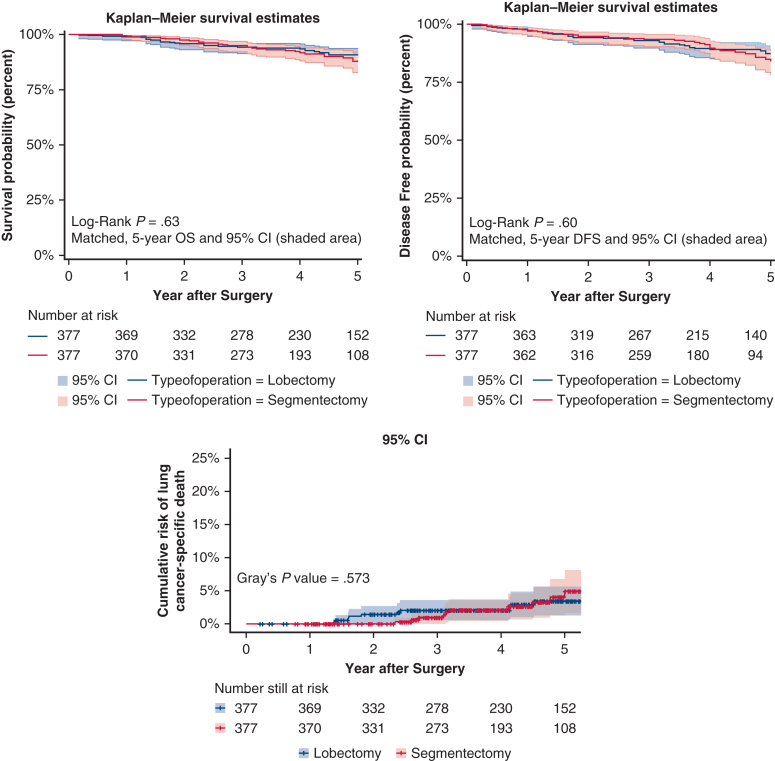
Overall survival, disease-free survival, and lung cancer–specific death after propensity score-matching in the entire dataset, with 95% CI.

Both in matched and unmatched cohorts, OS, DFS, and LCSD rates were similar between lobectomy and segmentectomy in entire dataset (Figure 1). In the matched cohort, 5-year OS rates were lobectomy 90.7% (95% CI, 87.4-93.7%) versus segmentectomy 87.7% (95% CI, 83.4-92.2%), *P* = .6; 5-year DFS rates were lobectomy 81.7% (95% CI, 77.3-86.4%) versus segmentectomy 77.6% (95% CI, 72.1-83.6%), *P* = .5; and 5-year LCSD rates were lobectomy 3.4% versus segmentectomy 4.7%, *P* = .6, which were similar. Proportional hazards assumption was not violated for survival (*P* = .6), recurrence (*P* = .8), or for LCSD (*P* = .8).

**Figure 2:**
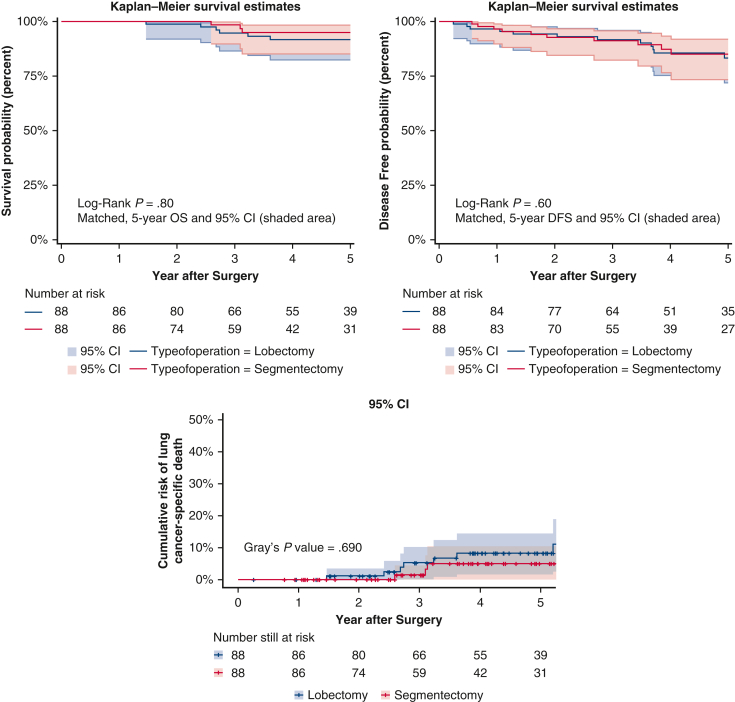
Overall survival, disease-free survival, and lung cancer–specific death after propensity score-matching in the aggressive dataset, with 95% CI.

The outcome was the same in the aggressive dataset (Figure 2). In its matched cohort, 5-year OS rates were lobectomy 88.0% (95% CI, 80.9-95.7%) versus segmentectomy 89.1% (95% CI, 82.2-96.6%), *P* = .8; 5-year DFS rates were lobectomy 79.8% (95% CI, 70.8-89.8%) versus segmentectomy 80.6% (95% CI, 71.6-90.6%), *P* = .6; and 5-year LCSD rates were lobectomy 6.0% versus segmentectomy 7.8%, *P* = .8, which were not statistically different. The proportional hazards assumption was not violated for survival (*P* = .1), recurrence (*P* = .8), or for LCSD (*P* = .3).

In parsimonious Cox regression analysis of entire dataset, the following variables significantly influenced OS: sex, age, comorbidities, surgical approach, clinical tumor size, pathologic tumor size, and SUVmax; DFS was only impacted by pathologic N, and LCSD was influenced by sex, clinical tumor size, pathologic N, aggressive dominant pattern, and pathologic tumor size (Table E2).

In the aggressive dataset, according to parsimonious model for Cox regression, no variables impacted OS; DFS was influenced by pathologic tumor size (HR, 2.18; 95% CI, 1.05-4.51), *P* = .03, adjuvant chemotherapy (HR, 0.8; 95% CI, 0.71-0.99), *P* = .04, and surgical approach (HR, 1.04; 95% CI, 1.02-1.05), *P* = .001. However, LCSD was impacted by sex (HR, 1.87; 95% CI, 1.18-2.95), *P* = .006, and SUVmax (HR, 1.25; 95% CI, 1.05-1.50), *P* = .01. Loco-regional recurrence is superior in segmentectomy group both in entire (Table E3) and aggressive datasets (Table E4), only in the matched cohort.

## Discussion

We demonstrated that OS, DFS, and LCSD were similar between lobectomy versus segmentectomy for invasive lung adenocarcinoma even for those with aggressive histologic patterns. In the unmatched cohort, loco-regional recurrence was similar between both groups in the entire dataset and lower in the segmentectomy group of the aggressive dataset; however, it was greater in segmentectomy group in the matching cohorts of both datasets. Aggressive histologic patterns only impacted LCSD and only when they were dominant.

We are in an area in which each lung adenocarcinoma nodule is expected to be systematically analyzed in its histologic, immunochemical, and genetic mutation aspects with substantial granularity.^2^ This requires a standardized assessment and implies personalized management of patients.

We had 30% of patients with aggressive histologic patterns, which seems slightly lower than the anticipated incidence.^4^^,^^5^ This is mainly because in almost all centers, histologic assessment was performed by more than 1 pathologist only in challenging cases. This hypothesis is supported by a substantially greater proportion (56.7%) of patients with a single pattern in our series, whereas it has been reported to be approximately 10%.^11^

There is a real need for each nodule to be systematically assessed either by at least 2 dedicated pathologists or by digital examination followed by a dedicated pathologist to ensure a correct identification of some patterns, such as micropapillary. Most importantly, this should help to avoid missing nondominant aggressive patterns.

In our series, histologic pattern was an independent prognostic factor only when dominant and for LCSD. In contrast, other papers^3^^,^^4^^,^^6^^,^^7^^,^^10^ reported that aggressive patterns were associated with poor OS and DFS, even when nondominant. This difference in our series may be explained by the fact that, most specimens were not systematically classified by at least 2 pathologists. In addition, we only included patients with smaller tumors. In our previous series, dominant pattern only impacted DFS.^8^ In the current study, the impact of dominant pattern on OS and DFS may be masked by other poor prognosis features such as SUV max, clinical tumor size, pathologic tumor size, and pathological N, which negatively influenced DFS or OS. Tsao and colleagues^12^ analyzed the impact of adenocarcinoma-dominant patterns in clinical trials where histologic classification was reviewed by 2 coauthors of the current classification. They found that aggressive-dominant patterns only impacted DFS and specific DFS.

Adjuvant chemotherapy positively impacted DFS in aggressive pattern dataset. In total, 18.2% of patients with aggressive pattern had systemic adjuvant chemotherapy, whereas 6.1% of patients with aggressive pattern had a nodal upstaging. This finding aligns with results of Tsao and colleagues^12^ and of previous study.^8^

Solid and micropapillary patterns are predictable and can be influenced by oncologic lung resection quality (lung resection with safe margins and systematic lymph node dissection). In a study conducted at the Memorial Sloan-Kettering Cancer Center, where the adenocarcinoma histologic classification was systematically performed by 2 pathologists, pioneers of the current classification. Nitadori and colleagues^7^ found that increasing of resection margins mitigated the poor effect of micropapillary pattern. In addition, they observed a reduction in the negative impact of micropapillary pattern in the lobectomy group, where lymph node dissection was by far systematically performed.

A National Cancer Database article analyzed data from 249,391 patients who underwent lobectomy or segmentectomy from 2004 to 2018. Logan and colleagues^13^ reported that segmentectomy performed after oncologic principles was likely performed in academic centers and was associated with an improved OS. However, only 12.6% of segmentectomies met all oncologic quality requirements, and only 27.4% of patients who underwent segmentectomy had ≥10 dissected lymph nodes. The low adherence to oncologic criteria could explain conflicting findings between studies, especially those that include patients with poor pathologic characteristics.

Pathologic N0 is challenging to evaluate, particularly in retrospective studies, and in patients who underwent sublobar resections, even when lymph node dissection was claimed systematic. Luo and colleagues^11^ evaluated the indication of completion lobectomy for nodal upstaging patients operated by segmentectomy from 2010 to 2020. They included only patients with reported lymph node dissection. Surprisingly, patients with pN0 (unlike those with pN1 and pN2) who underwent segmentectomy had a worse OS (compared with lobectomy) after 2 years. This raises questions of the systematic nature of lymph node dissection in patients who undergo segmentectomy. This concern is further emphasized by the greater prevalence of upstaging N1 and N2 in the lobectomy group. In addition, it's worth noting that segmentectomies were more performed by video-assisted thoracic surgery or robotic-assisted thoracic surgery,^11^ which can be understandable in a period when efficiency of minimally invasive thoracic surgery raised concerns about quality of systematic lymph node dissection and segmentectomy. In our series, a greater prevalence of patients with aggressive patterns received adjuvant systematic treatment, which may improve oncologic outcome in those who underwent segmentectomy. In addition, some of our centers systematically performed lymph node and margins frozen section in patients operated on by segmentectomy.

Like for these adenocarcinoma histologic aggressive patterns,^8^ oncologic segmentectomy (with safe margins and lymph node dissection) has been proven to be efficient for patients with early-stage lung cancer and with clinically aggressive features,^14^ visceral pleural invasion,^15^, ^16^, ^17^ spread through air spaces,^11^^,^^18^ lymphovascular invasion,^19^ and even those with nodal upstaging.^11^^,^^19^ Surgical resection remains the cornerstone option for both diagnosing and treating early-stage lung cancer. Oncologic segmentectomy has proven its efficacy even in case of aggressive features.

In addition to its retrospective nature, this study presents other limitations. Histologic classification was determined by 1 pathologist in most cases. Margins were not measured for all patients, comparison was not adjusted by location, and center effect was not objectively assessed. However, its multicentric setting of a clinical standard practice seems reassuring and it can be used as a basement for further investigations.

## Conclusions

Oncologic segmentectomy was comparable to lobectomy for patients with cT1a-bN0M0 invasive lung adenocarcinoma, even for those with micropapillary or solid patterns.

## Conflict of Interest Statement

The authors reported no conflicts of interest.

The *Journal* policy requires editors and reviewers to disclose conflicts of interest and to decline handling or reviewing manuscripts for which they may have a conflict of interest. The editors and reviewers of this article have no conflicts of interest.

## Figures and Tables

**Table E1:** Follow-up by number of outcomes/100 patients-year

Group	Entire dataset			Aggressive dataset		
	PTFR survival	PTFR recurrence	PTFR LCSD	PTFR survival	PTFR recurrence	PTFR LCSD
Before PSM						
All data sets	5.69	3.78	2.41	6.32	6.54	3.94
Lobectomy	6.03	3.70	2.44	6.52	6.92	3.98
Segmentectomy	4.86	5.24	2.49	7.21	5.90	4.12
After PSM						
All data sets	5.35	4.01	2.43	6.77	5.47	3.97
Lobectomy	6.27	3.74	2.42	6.80	6.33	4.66
Segmentectomy	4.86	5.24	2.49	7.21	5.90	4.12

*PTFR*, Person-time follow-up; *LCSD*, lung cancer–specific death; *PSM*, propensity-score matching.

**Table E2:** Cox regression multivariable analysis in the entire dataset

Variables	HR	95% CI	*P* value
Survival			
Sex	1.56	1.15-2.11	.004
Age	1.02	1.00-1.04	.006
PS	1.06	0.80-1.39	.7
Comorbidities	1.10	1.01-1.20	.01
Pack-year	1.25	0.98-1.61	.07
Approach	1.05	1.00-1.10	.02
Clinical tumor size	1.78	1.19-2.66	.005
Pathologic tumor size	1.90	1.22-2.96	.005
Nondominant	0.93	0.77-1.12	.4
SUVmax	1.16	1.01-1.33	.03
Recurrence			
Smoking status	1.10	0.85-1.44	.4
Comorbidities	1.04	0.95-1.13	.4
Approach	1.01	0.98-1.05	.5
SUVmax	0.95	0.82-1.10	.5
Pack year	1.16	0.87-1.56	.3
Pathologic N	1.34	1.02-1.79	.04
Lung cancer–specific death			
Sex	1.94	1.17-3.2	.01
PS	1.33	0.84-2.10	.2
Pack Year	1.29	0.84-1.98	.2
Clinical tumor size	2.01	1.03-3.94	.04
Pathologic tumor size	2.44	1.21-4.94	.01
Pathologic N	1.97	1.31-2.97	.001
Dominant	0.48	0.32-0.71	<.001
SUVmax	1.21	0.98-1.50	.08

*HR*, Hazard ratio; *PS*, performance status; *SUVmax*, maximum standardized uptake value.

**Table E3:** Linearized risk for recurrences and second primary lung cancer in the entire dataset

Recurrence & second primary	Unmatched cohort		Matched cohort	
	Lobectomy n = 1029	Segmentectomy n = 377	Lobectomy n = 377	Segmentectomy n = 377
Recurrence site				
Loco-regional	0.078	0.073	0.048	0.076
Distant	0.071	0.056	0.048	0.041
Locoreg-distant	0.017	0.022	0.039	0.000
Second primary	0.007	0.013	0.010	0.015

**Table E4:** Linearized risk for recurrences and second primary lung cancer in the aggressive dataset

Recurrence & second primary	Unmatched cohort		Matched cohort	
	Lobectomy n = 339	Segmentectomy n = 88	Lobectomy n = 88	Segmentectomy n = 88
Recurrence site				
Loco-regional	0.036	0.011	0.005	0.036
Distant	0.018	0.003	0.002	0.009
Locoreg-distant	0.003	0.004		
Second primary	0.014	0.016	0.044	0.040
